# Supplementary material for: CFTR Rescue by Lumacaftor (VX-809) Induces an Extensive Reorganization of Mitochondria in the Cystic Fibrosis Bronchial Epithelium
Source: Cells. 2022 Jun 16;11(12):1938. doi: 10.3390/cells11121938 (PMC9222197; doi:10.3390/cells11121938)

Figure S1

A) Principal Component Analysis of the SWATH proteomics dataset.

B) Changes in protein expression in VX-809 Vs DMSO treated cells. Each feature on x axis represents a protein. P values (y axis) are generated by Student's t test and corrected for multiple testing. At a threshold of p>0.05 there are no significant changes in protein expression were found (all features are reported in grey).

C) CFTR expression following VX-809 treatment is not significantly increased (p=0.17 in an unpaired t-test).

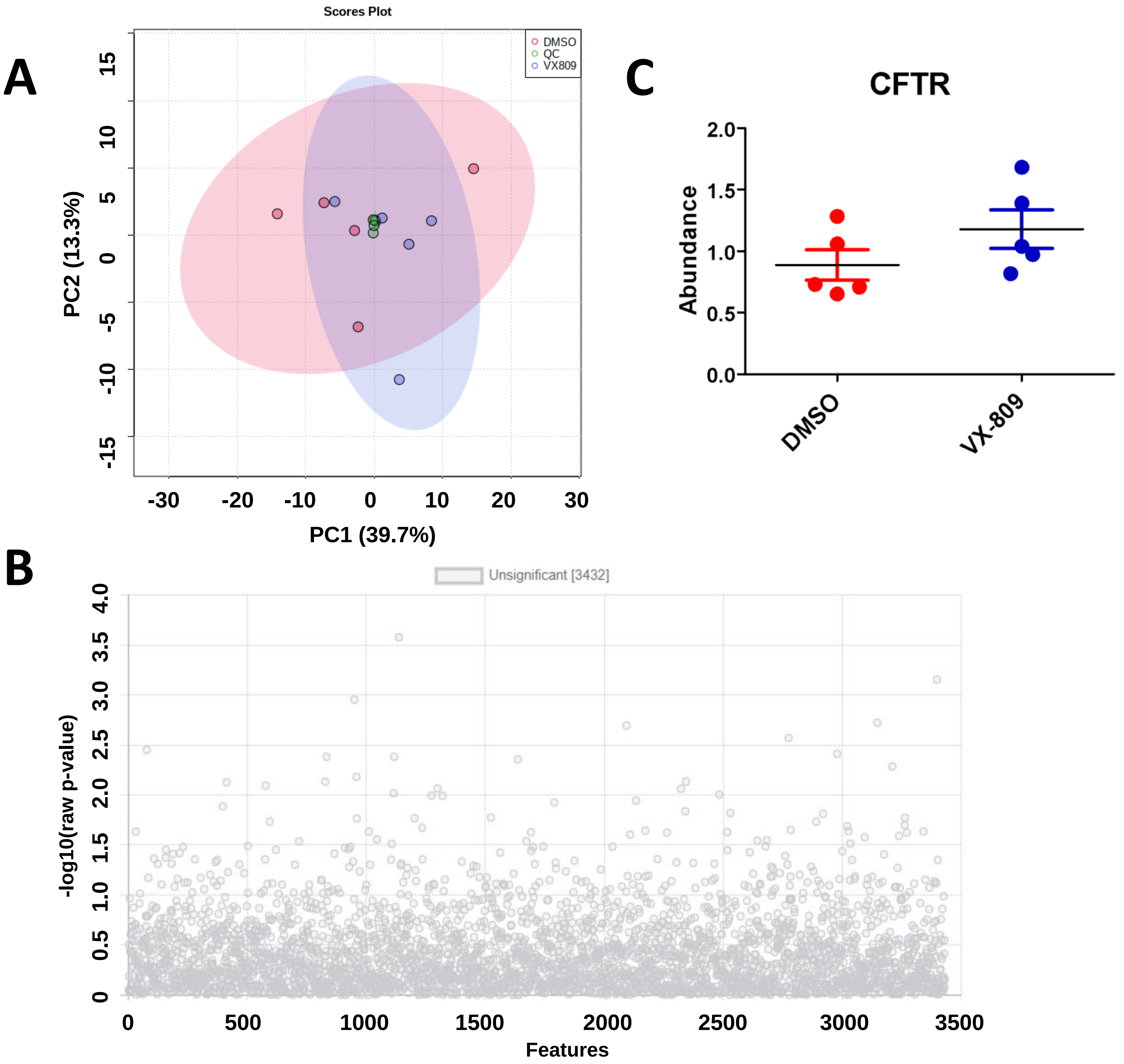

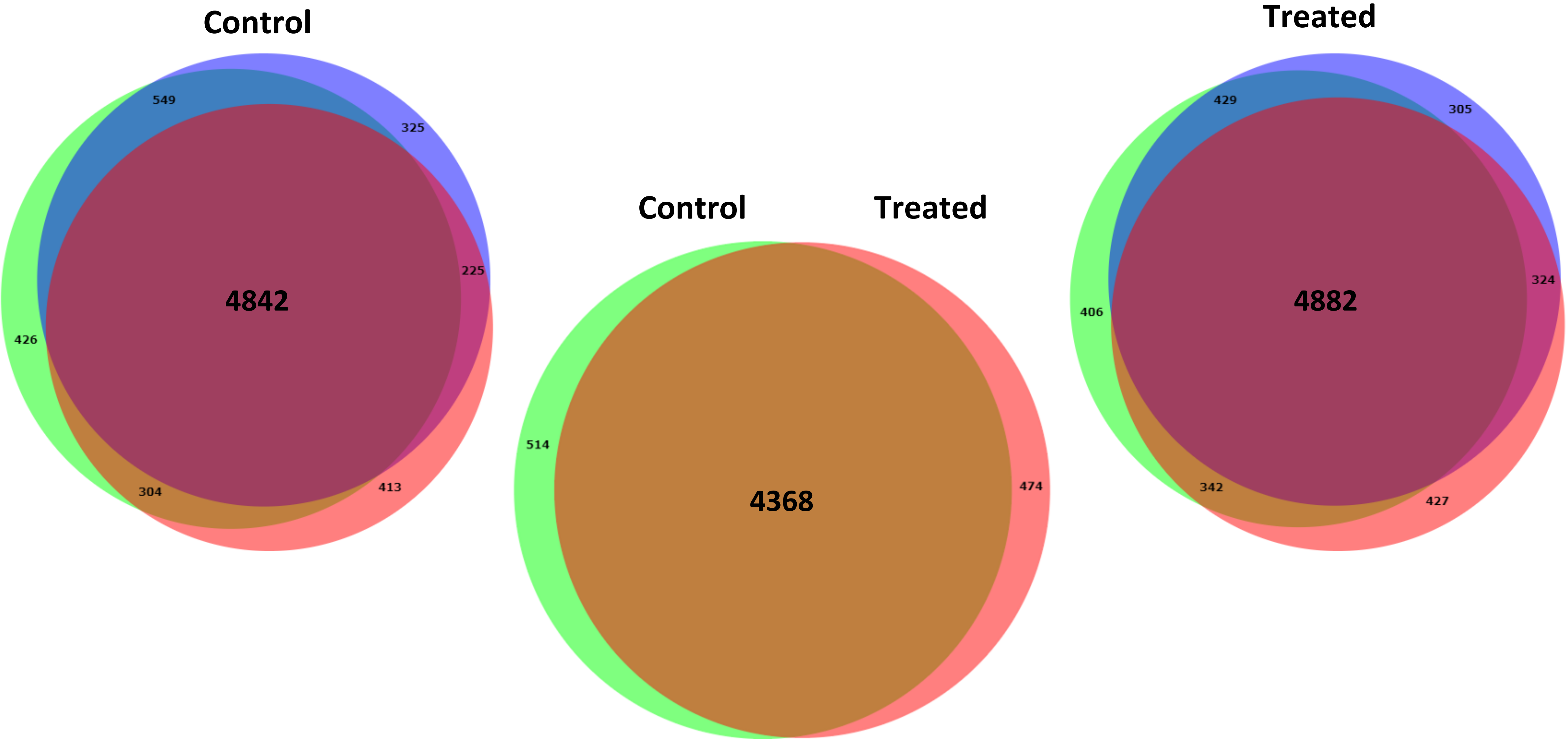

Figure S2
Venn diagrams for the identified proteins in the Control and Treated groups, for the three replicates. Central Venn represents the overlap of identified proteins in common between the two groups.

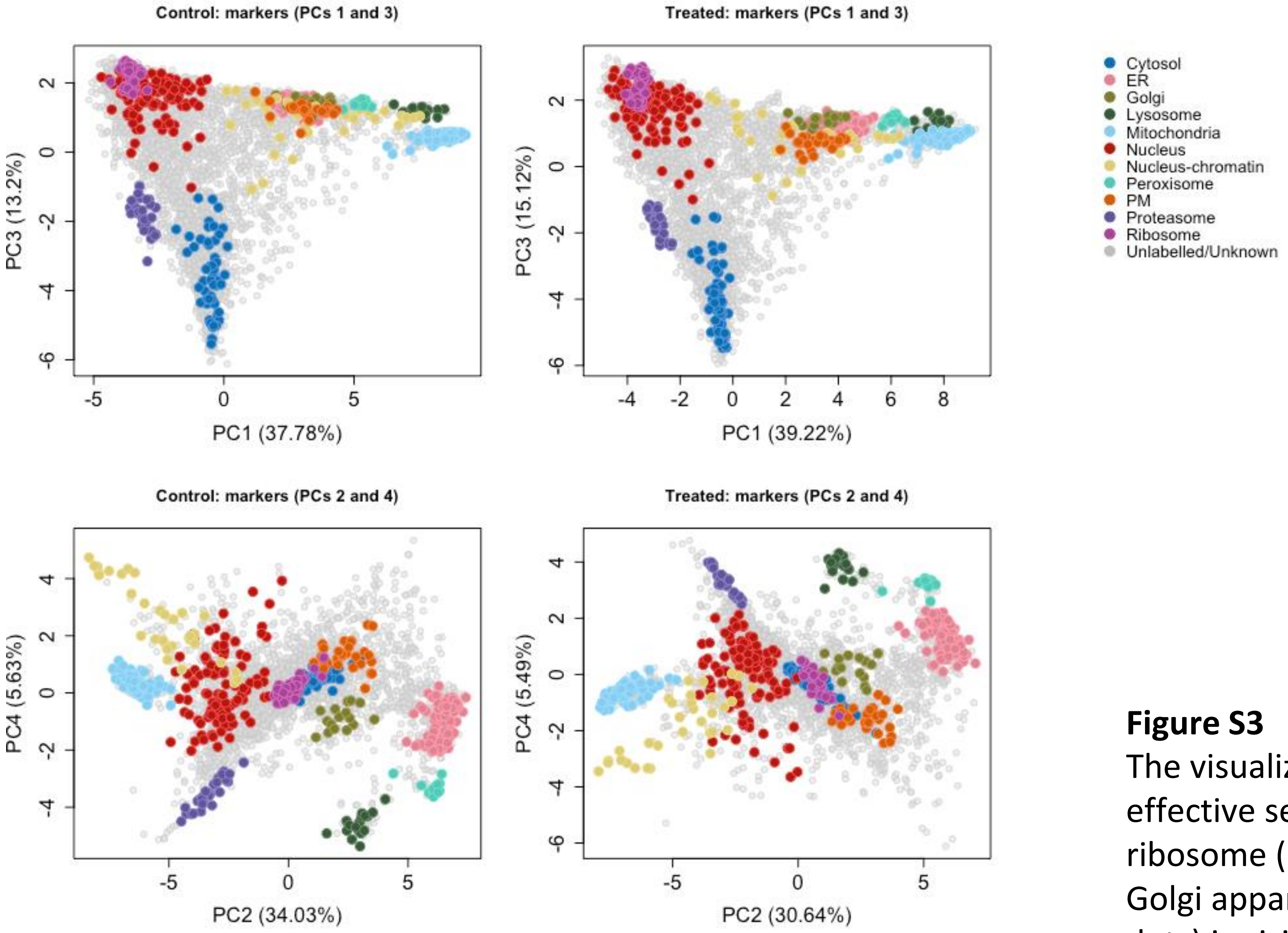

Figure S3
The visualization as PC1 Vs PC3 shows the effective separation of nucleus (red dots) and ribosome (purple dots), while the separation of Golgi apparatus (green dots) and PM (orange dots) is visible over PC2 and PC4.

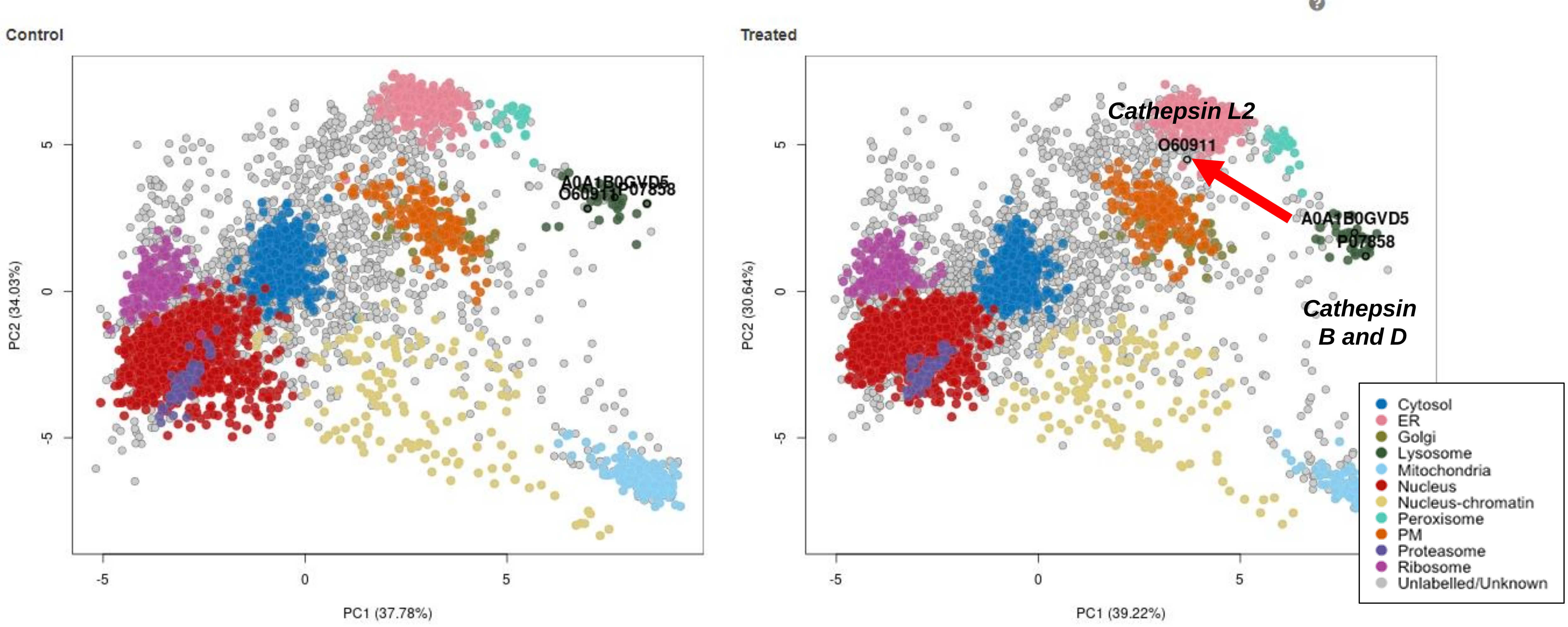

Figure S4

Specific relocalization of Cathepsin L from lysosome to the ER, highlighted by LOPIT-DC data. Cathepsin L (ID 060911) is moving from the green area (lysosome) to the pink area (ER proteins)

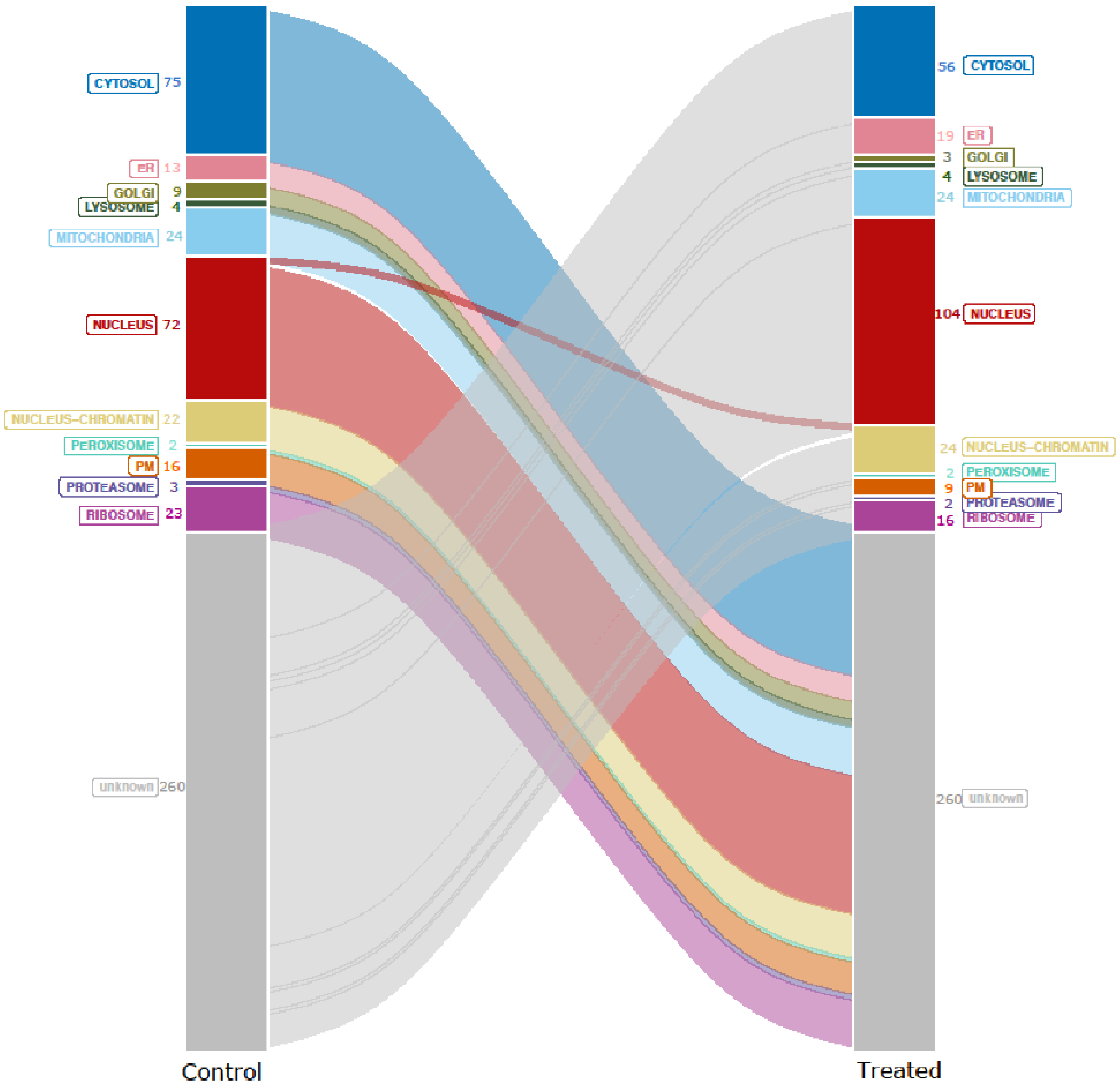

Figure S5: Alluvial plot representing the spatial re-localization of proteins after treatment with VX-809.

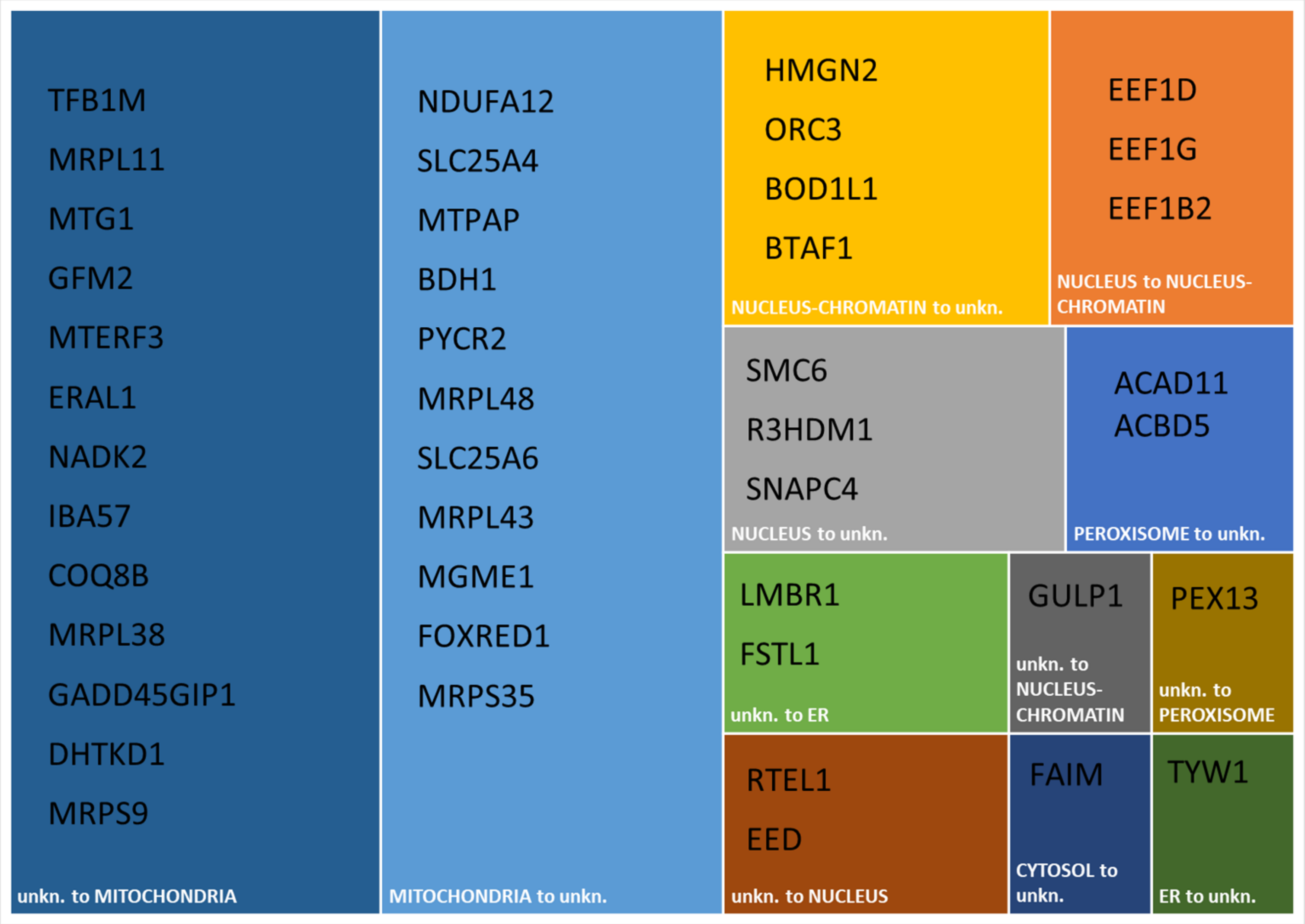

Figure S6: Spatial reorganization of 45 proteins that change their localization in association with VX-809

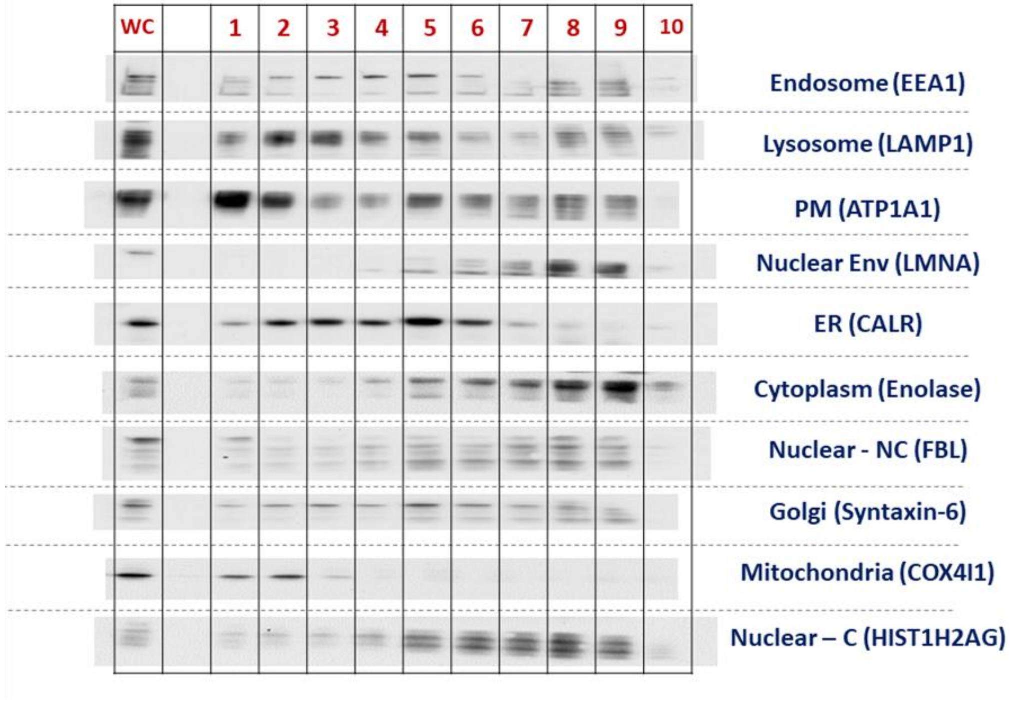

Figure S7: Wester-Blot analysis of the protein content of CFBE41o-, fractionated by differential centrifugation. The abundance profile of 10 organelle-specific marker proteins (listed on the right) was detected across the collected fractions to evaluate the efficiency in organelle separation. As a positive control the whole cell fraction (WC) was loaded together with all the fractions obtained by the differential centrifugation.

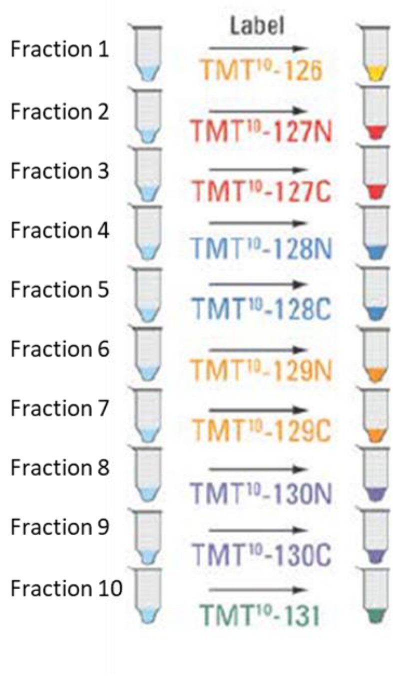

Figure S8: Scheme of the TMT labelling tags used for each fraction of the LOPIT-DC workflow.

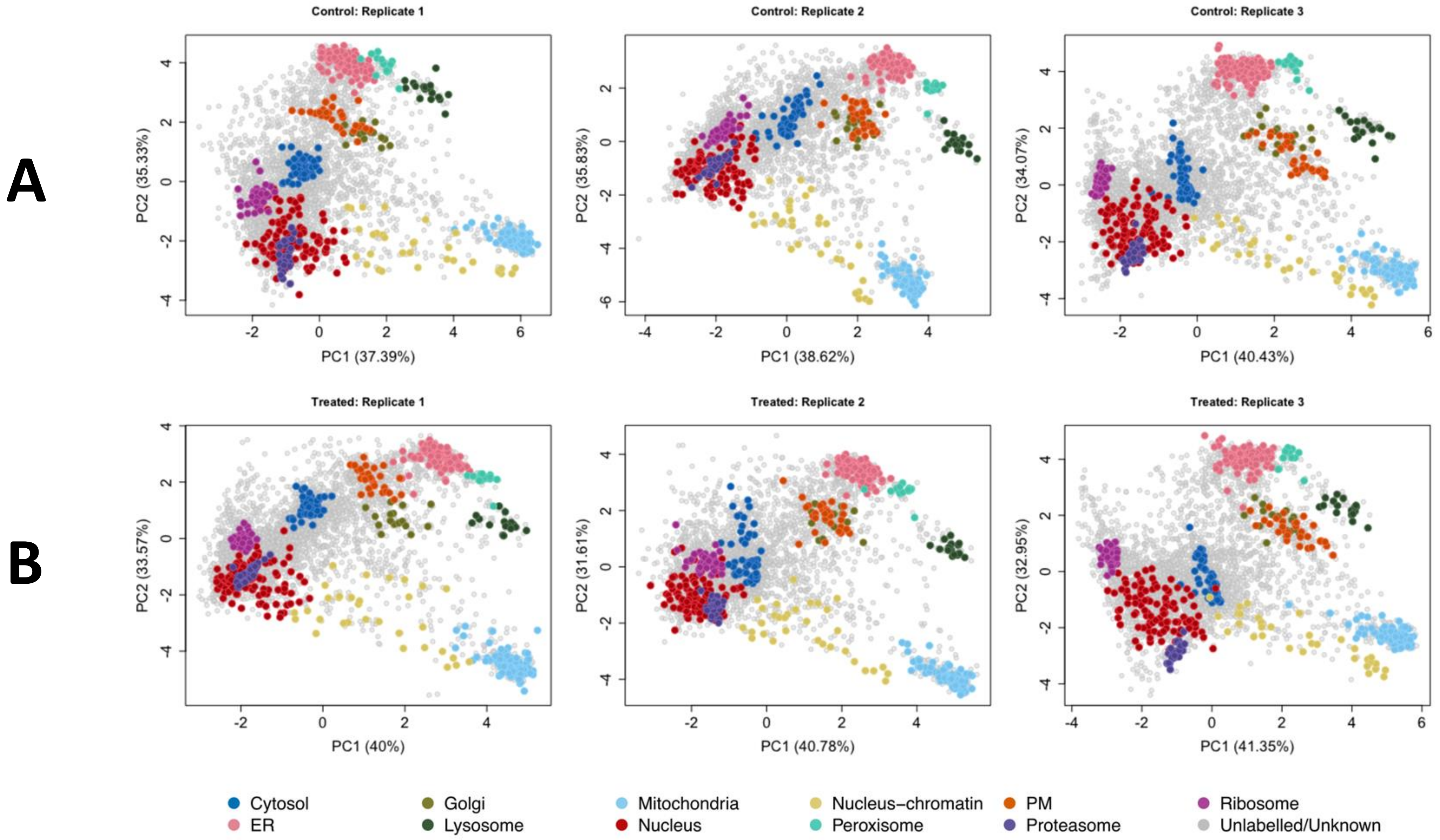

Figure S9

PCA cellular localization maps for the three individual replicates for the control (A) and VX-809 (B) groups.

Figure S10

Distribution profiles across the subcellular fractions collected by differential centrifugation of proteins classified to 11 subcellular localizations after applying SVM classification, with a 5% FDR cut-off. These profiles represent the distribution of protein abundances after each TMT 10- plex for each replicate has been combined (to give a total of 30 channels) for both control and treated conditions. Distribution profiles of proteins which fell below the 5% FDR cut-off are also included (unlabelled/unknown)."

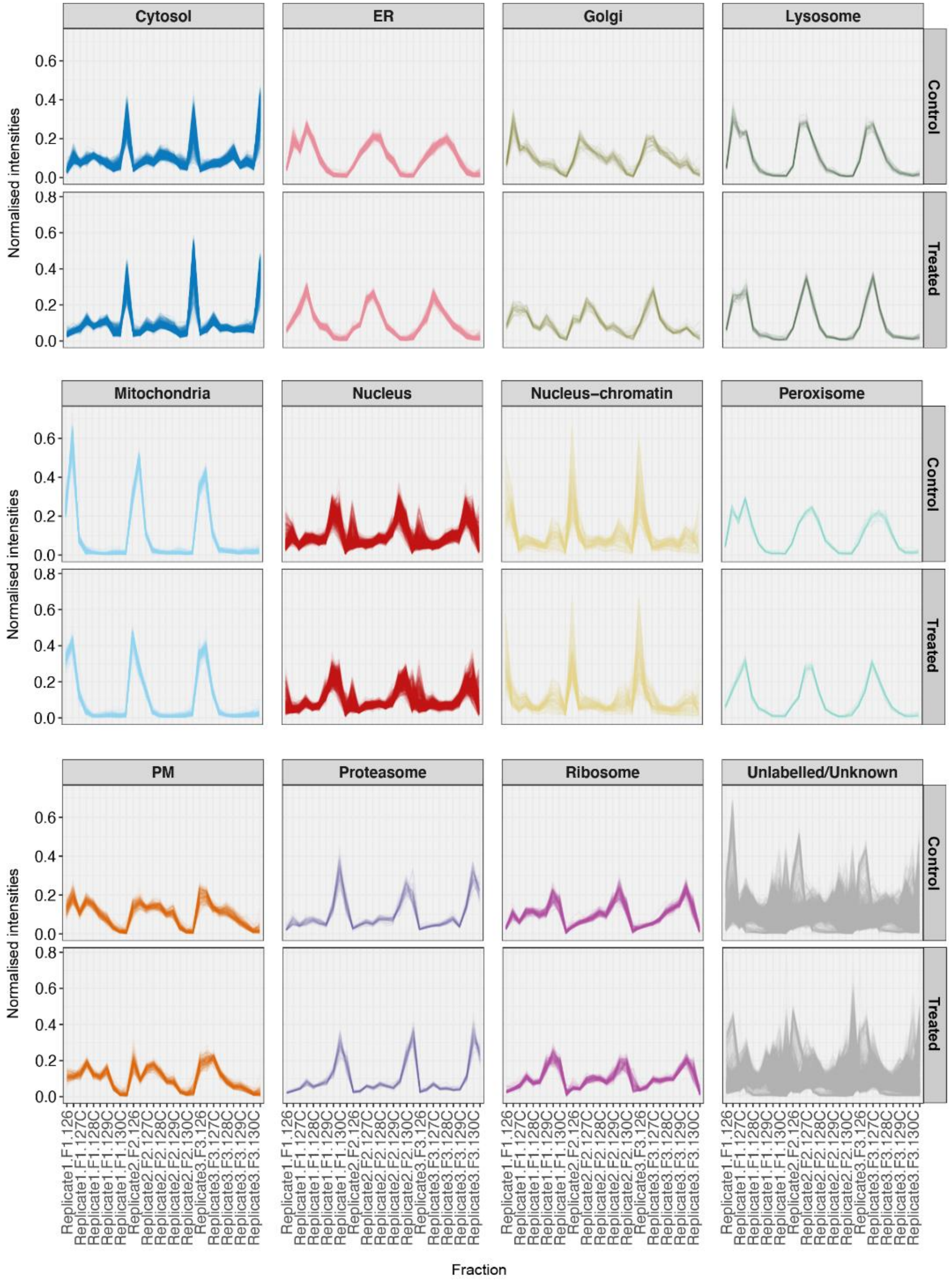

Supplement: Supplementary file 1 [file cells-11-01938-s001.zip › Supplementary Figures_Revised.pdf]
